# Porous Deproteinized Natural Rubber Film Loaded with Silver Nanoparticles for Topical Drug Delivery

**DOI:** 10.3390/pharmaceutics15112603

**Published:** 2023-11-08

**Authors:** Wiwat Pichayakorn, Pattwat Maneewattanapinyo, Chaowalit Monton, Nattakan Dangmanee, Jirapornchai Suksaeree

**Affiliations:** 1 Department of Pharmaceutical Technology, Faculty of Pharmaceutical Sciences, Prince of Songkla University, Hat-Yai, Songkhla 90112, Thailand; 2 Department of Pharmaceutical Chemistry, College of Pharmacy, Rangsit University, Muang, Pathum Thani 12000, Thailand; 3 Drug and Herbal Product Research and Development Center, College of Pharmacy, Rangsit University, Muang, Pathum Thani 12000, Thailand; 4 Faculty of Agro and Bio Industry, Cosmetic Technology and Dietary Supplement Products Program, Thaksin University, Ban Pa Phayom, Phatthalung 93210, Thailand

**Keywords:** porous deproteinized natural rubber film, silver nanoparticles, topical film

## Abstract

The work demonstrated the use of natural rubber for topical drug delivery. The first objective was to fabricate a porous deproteinized natural rubber film loaded with silver nanoparticles. Characterizing and assessing its formulation was the second objective. Surface pH, mechanical properties, swelling ratio, erosion, moisture vapor transmission rate, scanning electron microscopy/energy dispersive X-ray analysis, and X-ray diffraction were evaluated. In vitro studies and antibacterial activity were assessed. It was discovered that silver nanoparticles could enter the film and that their concentrations ranged between 7.25 and 21.03 µg/cm^2^. The pH of the film’s surface was 7.00. The mechanical properties of the film with silver nanoparticle loading differed from the blank film. After adding silver nanoparticles, the film eroded faster than before, but the swelling ratio was not affected significantly. Increased time utilization had an impact on the moisture vapor transmission rate of the film. Silver nanoparticles released easily from the film while there was less permeability. The dead pig-ear skin had significant silver nanoparticle accumulation. Potent antibacterial activity was seen in the film containing silver nanoparticles. The silver nanoparticle-loaded film may be used as a wound dressing for a topical film that promotes wound healing while also protecting the area from infection.

## 1. Introduction

Scientific study and developments in technology have combined recently to improve the field of wound healing and offer novel approaches that have the potential to completely change patient treatment [1,2,3]. Silver nanoparticles are attracting a lot of attention as an innovative therapeutic strategy for accelerating the healing of wounds due to their distinct physicochemical characteristics and exceptional antibacterial powers [4,5,6,7].

A film dressing is one of the simplest and most popular methods of treating wounds because it can be used as a physical barrier to cover a variety of superficial wounds, including burns, skin wounds, wounds caused by trauma or surgery, and infected wounds, while also providing a moist and secure environment for healing. It can attach to the wound site without really adhering to the wound edges [8]. The film dressing containing silver nanoparticles may be simply made by utilizing ethyl cellulose as a film. The film can control the release of silver nanoparticles at 102.98 ± 4.11% over 12 h and demonstrates antibacterial properties [7]. The biomaterial nanocomposite film for transdermal delivery is made by microwave irradiation followed by grafting of pectin with the copolymer of 2-acrylamido-2-methyl-1-propanesulfonic acid and acrylamide in the presence of silver nanoparticles. The nanocomposite film has high antibacterial action against *Staphylococcus aureus* and *Escherichia coli*. These findings demonstrate that the nanocomposite film is suitable for transdermal application, preventing contamination from skin contact occurring with perspiration and moisture [4]. Silver nanoparticle-loaded chitosan-starch-based film has been produced by first reducing silver nitrate in a chitosan solution with γ-ray irradiation and then casting it on a petri dish using a solution-casting process. Silver nanoparticles increase the tensile and oxygen gas barrier characteristics of polysaccharide-based films, as well as their antibacterial activities. The findings suggest that silver nanoparticle-loaded chitosan-starch-based films may be utilized as antibacterial materials in biomedical applications [9]. In addition, the antibacterial activity of silver nanoparticles can be improved by synthesizing them using *Ganoderma applanatum* and then loading them into topical formulations. The created film is acceptable for topical treatments and may be used in the development of possible future therapeutic agents for wound healing [5,6]. Thus, recent developments in biomaterials and regenerative medicine have demonstrated great potential for manufacturing the ideal wound dressing material, which can be applied easily, protect the wound’s surface from the outside environment, regulate the wound’s microenvironment, prevent inflammation, promote revascularization, and shorten the time it takes for a wound to heal [8,10,11].

Deproteinized natural rubber films have been investigated as potential materials for drug delivery systems due to their biocompatibility, flexibility, and simplicity of manufacturing. Drugs can be released under control over time using films made of deproteinized natural rubber. The permeability and rate of film dissolution may be adjusted to regulate the rate at which the drug is released. The film’s composition and thickness are modified to achieve this [12,13,14,15].

In this situation, an innovative device for the topical administration of drugs has been developed by the combination of porous deproteinized natural rubber films with silver nanoparticles. The porous deproteinized natural rubber-silver-nanoparticle film may be placed directly on the wound site in applications for wound healing. The silver nanoparticles are released when the film degrades over time, assisting in the healing process. The creation, fabrication, and characterization of these composite films are examined in detail in this research, along with how they may affect the way drugs are administered transdermally. The characteristics included surface pH, mechanical properties, swelling ratio, erosion, moisture vapor transmission rate, scanning electron microscopy/energy dispersive X-ray analysis (SEM/EDX), and X-ray diffraction (XRD). Evaluations of the in vitro studies and antibacterial activities were made.

## 2. Materials and Methods

### 2.1. Preparation of Deproteinized Natural Rubber Latex

As previously mentioned, deproteinized natural rubber latex was made in-house using an enzyme treatment and centrifugation technique that was safe for skin contact [16,17,18]. In brief, fresh natural rubber was deproteinized with 0.2 parts of alcalase enzyme per hundred of rubber, control pH 7–8 with 0.01 N sodium hydroxide solution, 1% *w*/*v* sodium dodecyl sulfate as a stabilizer, and 2% *w*/*v* Uniphen P-23 as a preservative and incubated for 48 h at 37 ± 2 °C. The washed natural rubber latex was centrifuged to separate it, then it was redistributed in distilled water and stored in the refrigerator at about 4 °C until it was used.

### 2.2. Preparation of Silver Nanoparticles

A chemical reduction technique was used to create a solution of colloidal silver nanoparticles, according to a previously described procedure [7]. In summary, the stock solution was created by combining two aqueous solutions: (I) an aqueous solution of 0.094 M silver nitrate stabilized with soluble starch, and (II) an aqueous solution of 0.07 M sodium borohydride reducing agent with soluble starch solution as a solvent. After that, the sodium borohydride stock solution was vigorously stirred while drops of the silver nitrate stock solution were added. The solution became a light-yellow color once the reaction was finished. The NanoPlus-3 (Micromeritics, Particulate Systems, Boulder, CO, USA) was used to assess particle size and PdI after it had been diluted 10 times with ultrapure water. The particle size of the sample was assessed using dynamic light scattering. Ten measurements were taken for the sample.

### 2.3. Preparation of Porous Deproteinized Natural Rubber Film Loaded with Silver Nanoparticles

The porous deproteinized natural rubber film was immersed in solutions containing different concentrations of silver nanoparticles for 24, 48, and 72 h. The porous deproteinized natural rubber film was immersed in a solution of silver nanoparticles. After that, the swelled film was dried again overnight in a hot air oven at 50 °C.

### 2.4. Determination of the Content of Silver Nanoparticles in Porous Deproteinized Natural Rubber Film

The calibration curve for the silver nanoparticle solution was produced by diluting pure silver nanoparticle solution with distilled water at eight concentrations ranging from 5 to 80 g/mL. After extracting the silver nanoparticles from the porous deproteinized natural rubber film with distilled water, the content of silver nanoparticles was measured using the UV/Visible spectrophotometer (UV-1800, Shimadzu Corporation, Kyoto, Japan). The transparent light-yellow film, with a thickness of about 220 µm, was prepared into squares of 1 cm × 1 cm and then cut into small pieces. It was sonicated for 120 min. A 0.45 µm cellulose acetate membrane was used to filter the undissolved film sample. The content of extracted silver nanoparticles solutions was estimated by comparing the calibration curve of pure silver nanoparticle solution. Three replications of the sample were prepared.

### 2.5. Surface pH

The film was placed in a glass tube with 0.5 mL of distilled water for 1 h and allowed to swell. After a minute of equilibration, a combination glass electrode was dipped in solution and positioned in contact with the film’s surface, and the pH value was recorded. Three replications of the sample were prepared.

### 2.6. Mechanical Properties

Pieces of 1 cm × 6 cm were cut off for each film sample. The TA.XT Plus texture analyzer (Texture Technologies Corporation and Stable Micro Systems, Ltd., Surrey, ND, USA) was used to perform the mechanical testing. The applied tensile force as a loaded cell was 500 N, and the test cross-head speed was 10 mm/min. The length of the measuring instrument was 1 cm × 1 cm. This approach was used in our research [5,19]. Five replications of the sample were prepared.

### 2.7. Swelling Ratio and Erosion

The dry sample was cut into a 2 cm × 2 cm square, which was then weighed (W_I_). Each square sample was then placed in a petri dish and soaked for 24 h at 37 °C in distilled water. It was weighed repeatedly until a steady weight was achieved (W_S_). The increased weight of the sample (W_S_–W_I_) was divided by the beginning weight (W_I_) of the film before being soaked in distilled water and multiplied by 100 to achieve the percentage of swelling ratio. Five replications of the sample were prepared.

The swollen film was once again dried at 60 °C. It was weighed frequently until a constant weight was achieved (W_D_). The percentage of erosion was calculated by dividing the sample’s lost weight (W_I_–W_D_) by the film’s beginning weight (W_I_) before it was soaked in distilled water. Five replications of the sample were prepared.

### 2.8. Moisture Vapor Transmission Rate

Moisture vapor transmission rate is the amount of moisture that passes through a unit area of film in a given amount of time. The transmission cells were made of glass bottles with an identical volume and diameter. The cells received a full washing before being dried in an oven. Each glass bottle was then filled with distilled water and given an anhydrous calcium chloride 1 g treatment. A piece of adhesive tape was used to hold the film to the brim of the glass bottle. After being weighed (total weight of the glass bottle and a film; TW_B_), these transmission cells were placed in a desiccator with saturated potassium chloride solution to maintain 84% relative humidity before being transported to a hot air oven at 40 °C. The transmission cell was weighed (TW_A_) on the first, second, third, fourth, and fifth days. The sample’s lost weight (TW_B_–TW_A_) was divided by the evaporation area to obtain the moisture vapor transmission rate. Five replications of the sample were prepared.

### 2.9. Scanning Electron Microscopy/Energy Dispersive X-ray Analysis

Elemental information and particle morphology were assessed using scanning electron microscopy with energy-dispersive X-ray analysis (SEM-EDX). The X-ray analytical microscope (Horiba, XGT-5200WR, Tokyo, Japan) and field emission scanning electron microscope (FE-SEM) connected to an Everhart–Thornley detector (Apreo SEM, FEI, Brno-Královo Pole, Czech Republic) with resolution 1.0 nm @ 15 kV, 1.9 nm @ 1 kV were used to analyze the silver composition and morphology of the sample. The magnifications utilized were 5000× and 100,000×.

### 2.10. X-ray Diffraction

The crystallinity of the silver nanoparticles in the film sample was determined using XRD equipment (Empyrean, Panalytical, Almelo, The Netherlands). A generator operating voltage of 40 kV and an X-ray source current of 30 mA with a stepped angle of 0.026° (2θ)/s in the angular range of 5–90° (2θ) were employed.

### 2.11. In Vitro Release of Silver Nanoparticles from Porous Deproteinized Natural Rubber Film

Vertical Franz diffusion cells (EMFDC06, Orchid Scientific, Maharashtra, India) with an effective diffusion area of 1.77 cm^2^ were used for in vitro release. The film sample was cut into 2 cm × 2 cm squares. A dialysis cellulose membrane (MWCO: 3500 Da, CelluSep^®^ T4, Membrane Filtration Product, Inc., Seguin, TX, USA) was used; it was pre-soaked in a receptor medium overnight before being used. The receptor medium included 12 mL of an isotonic phosphate-buffered solution of pH 7.4 with a water jacket at a temperature of 37 ± 0.5 °C, and it was continually stirred at a speed of 600 rpm using a magnetic stirrer. One mL of the receptor medium was taken out at time intervals of 0.5, 1, 2, 4, 6, 8, 10, 12, and 24 h. This was then immediately replaced with an equivalent volume of fresh isotonic phosphate-buffered solution. The UV/Visible spectrophotometer (UV-1800, Shimadzu Corporation, Japan) was used for analyzing the composition of silver nanoparticles. It was operated at 470 nm at room temperature. The test was replicated three times.

The release kinetics of silver nanoparticles from porous deproteinized natural rubber film were studied using several types of mathematical models, including the zero-order, first-order, Higuchi, and Korsmeyer–Peppas models. The kinetic models based on the four models that follow Equations (1)–(4) were evaluated using DDSolver.

Zero-order model → *Q_t_* = *Q*_0_ + K_0_*t* (1)

First-order model → ln *Q_t_* = ln *Q*_0_ − K_1_*t* (2)

(3) $$\mathrm{Higuchi}’s\;\mathrm{model}\to Q_{t}=K_{H}\sqrt{t}$$

(4) $$\mathrm{Korsmeyer}–\mathrm{Peppas}\;\mathrm{model}\to \frac{Q_{t}}{Q_{0}}=K_{\mathrm{KP}}t^{n}$$

where *Q*_0_ was the amount of the initial silver nanoparticles and *Q_t_* was the amount of silver nanoparticles.

### 2.12. In Vitro Skin Permeation of Silver Nanoparticles from Porous Deproteinized Natural Rubber Film

Vertical Franz diffusion cells (EMFDC06, Orchid Scientific, Maharashtra, India) with an effective diffusion area of 1.77 cm^2^ were used for in vitro skin permeation. The film sample was cut in squares of 2 cm × 2 cm. Dead pig-ear skin was used as a partition; it was pre-soaked in a receptor medium overnight before being used. The receptor medium included 12 mL of an isotonic phosphate-buffered solution of pH 7.4 with a water jacket at a temperature of 37 ± 0.5 °C, continually stirred at a speed of 600 rpm using a magnetic stirrer. One mL of the receptor medium was taken out at time intervals of 0.5, 1, 2, 4, 6, 8, 10, 12, and 24 h. This was then immediately replaced with an equivalent volume of fresh isotonic phosphate-buffered solution. The UV/Visible spectrophotometer (UV-1800, Shimadzu Corporation, Kyoto, Japan) was used for analyzing the composition of silver nanoparticles. It was operated at 470 nm at room temperature. The sample was replicated three times.

At the finish of the in vitro skin permeation experiment, the dead pig-ear skin samples were removed from the diffusion cells and twice rinsed with 1 mL of distilled water to eliminate any remaining residue from the skin’s surface. Each piece of skin was cut into smaller pieces before being homogenized and sonicated for 60 min to extract the substance in distilled water. The supernatant from centrifuged samples was collected and filtered with 0.45 µm, and then the samples were examined with a UV/Visible spectrophotometer.

### 2.13. Antimicrobial Activity Evaluation

The agar diffusion technique was used to evaluate the antibacterial activity of the chosen porous deproteinized natural rubber film loaded with silver nanoparticles. The antibacterial activity of the porous deproteinized natural rubber film loaded with silver nanoparticles was evaluated using the disk diffusion test. The antibacterial activity evaluation used the typical microorganisms: *Staphylococcus aureus* and *Staphylococcus epidermidis*. The film sample was then divided into discs of 6 mm in diameter and contained silver nanoparticles. The film sample was carefully positioned on the colonized agar plate. A cellulose disc filled with silver nanoparticles was used as a positive control. The plate was incubated for 24 h at 37 °C. The bacterial growth inhibition zone was measured on a millimeter scale and the results were presented as mean ± S.D. The sample was replicated three times.

### 2.14. Statistic Analysis

The average findings for each experiment were subsequently calculated and given as the mean standard ± deviation value. All results were statistically analyzed using one-way analysis of variance, followed by post hoc analysis (to compare the difference across several data sets) for significance at *p* < 0.05.

## 3. Results and Discussion

### 3.1. Determination of the Content of Silver Nanoparticles in Porous Deproteinized Natural Rubber Film

The color of the colloidal silver nanoparticle solution after preparation was yellow-brown, suggesting that the silver nanoparticles were successfully formed. The particle size and PdI values were 102.43 ± 8.54 nm and 1.54 ± 0.34, respectively. The hydrodynamic diameter (hydrodynamic radius of particles) of the silver nanoparticles was calculated, suggesting that agglomerates were ideally recognized via dynamic light scattering. The hydrodynamic dimension of the main particles or agglomerates in the liquid is crucial in defining the behavior of nanoelements in a fluid [20]. Consequently, the estimated particle size may be bigger than that obtained by other techniques of measurement. UV/Visible spectroscopy was employed to investigate the biosynthesis of silver nanoparticles. This approach is ideal for identifying surface plasmon resonance peaks. When silver nanoparticles are in the transmission band, they display surface plasmon resonance peaks created by silver electrons vibrating in resonance with a certain wavelength of the incoming light [21]. The presence of the highest absorption band at 470 nm for the silver nanoparticle solution, which corresponds to the common surface plasmon band of silver nanoparticles, proved the effective synthesis of silver particles. The calibration curve for silver nanoparticles was created in eight concentrations ranging from 5 to 80 g/mL. The equation was y = 0.0116x + 0.0029, and the linear regression coefficient (R^2^) was 1.000.

There has been a lot of research carried out on polymer and hydrogel silver nanocomposites for use as antibacterial materials. However, these systems can only release silver nanoparticles from their swelling polymer/hydrogel networks in aqueous fluids and not in dry conditions [22,23,24]. Basically, both the silver nanoparticles and the deproteinized natural rubber were dispersed in distilled water. Consequently, deproteinized natural rubber latex and a silver nanoparticle solution might be mixed to create a film, which was subsequently formed by evaporating the distilled water. However, when a solution of silver nanoparticles was added to deproteinized natural rubber latex, rubber particles began to accumulate. Therefore, the film could not be made. Due to this limitation, the deproteinized natural rubber latex was therefore first generated as a film, and then the film became immersed in the silver nanoparticle solution. Silver nanoparticles might then be inserted into a film formation after the deproteinized natural rubber film swelled to create a porous surface. 

In order to choose the best conditions for preparation, the content of silver nanoparticles in the porous deproteinized natural rubber film was first observed. When the film swelled, it became porous in its film layer, allowing the silver nanoparticles to enter the structure. Depending on the increasing concentration of the silver nanoparticle solution used to immerse the film, the amount of silver nanoparticles detected in the porous deproteinized natural rubber film ranged from 7.25 to 16.97 g/cm^2^, as shown in Table 1. In addition, the amount of silver nanoparticles tended to increase when the film’s immersion period was prolonged. When it was extended to 72 h, however, the amount of silver nanoparticles detected in the porous deproteinized natural rubber film showed no significant difference from the amount after 48 h of immersion. Therefore, the PN04 was the appropriate and best preparation for the porous deproteinized natural rubber film loaded with silver nanoparticles for use in future evaluations.

### 3.2. Surface pH

During the manufacturing process of the deproteinized natural rubber latex, the pH was adjusted to 7–8 using a 0.01 N sodium hydroxide solution because this pH value is most effective for the process of alcalase enzyme digesting proteins in natural rubber latex [16,17,18]. Consequently, when the deproteinized natural rubber latex was produced as a film, the prepared film’s surface pH was 7.22 ± 0.06. The surface pHs of all porous deproteinized natural rubber film loaded with silver nanoparticles were about 7.00 after the produced deproteinized natural rubber film was immersed in the solution containing silver nanoparticles to entrap the silver nanoparticles into its structure (Table 1). This happened because of the produced deproteinized natural rubber film’s pH being diluted during the immersion process. Normal skin has a pH between 4.0 and 7.0, which is slightly acidic and acts as a barrier against bacteria, viruses, and other harmful pollutants [25]. When the formulation comes into contact with the skin, the pH of the formulation should be compatible with the pH of normal skin, which ranges from 4.0 to 7.0 [26]. Therefore, the surface pH responsiveness of all films was investigated and was approved as safe for contact with the skin without irritation.

### 3.3. Mechanical Properties

**Figure 1:**
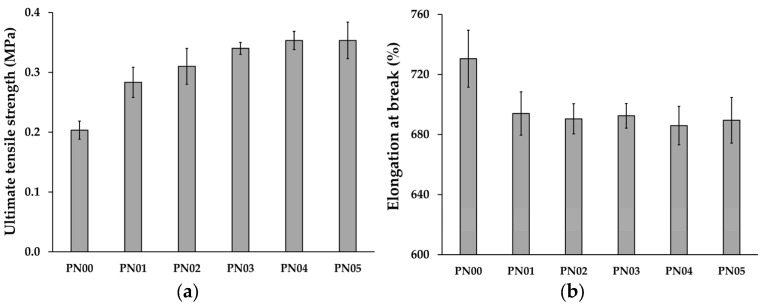
Mechanical properties of porous deproteinized natural rubber film loaded with silver nanoparticles: (**a**) ultimate tensile strength and (**b**) elongation at break.

Figure 1 presents the mechanical properties of porous deproteinized natural rubber film loaded with silver nanoparticles. The mechanical properties of the deproteinized natural rubber film demonstrated a low ultimate tensile strength and a high elongation at break, indicating a soft and flexible film. This result is related to the previous publication [18], Pichayakorn, W., and a co-worker reported that the deproteinized natural rubber film can produce an excellent elastic film, but it has poor adhesive characteristics, including low peel strength and tack adhesion. Blending deproteinized natural rubber with a variety of types and amounts of polymer and plasticizer can result in suitable films with varying mechanical characteristics, as well as an improvement in poor mechanical properties. 

The ultimate tensile strength of a porous deproteinized natural rubber film loaded with silver nanoparticles increased while the percentage elongation at break decreased. This might be due to the possibility of silver nanoparticles inserting themselves into the film fabrication, reducing the flexibility of the film. It would be obvious that as the amount of silver nanoparticles in the porous deproteinized natural rubber film increased, the film’s strength increased but its elongation decreased. However, all preparations demonstrated that the film was flexible enough to stay in contact with the skin. Dias Murbach, H. and a co-worker considered the changes in the behavior of natural rubber latex film after loading ciprofloxacin. The addition of ciprofloxacin to the natural rubber latex membrane affected its mechanical properties. The new material stiffened and became brittle, with less plastic deformation and less ductility [27].

### 3.4. Swelling Ratio and Erosion

**Figure 2:**
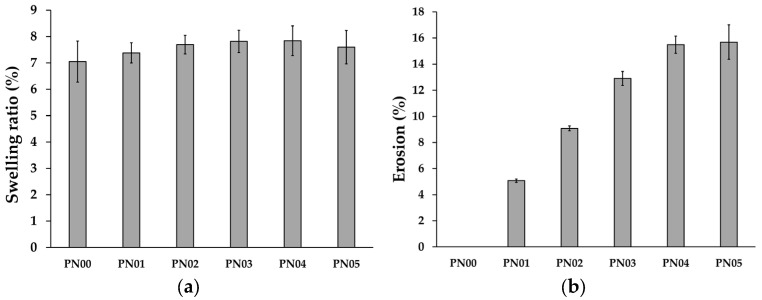
(**a**) Swelling ratio and (**b**) erosion of porous deproteinized natural rubber film loaded with silver nanoparticles.

The swelling capacity of the film plays an important role in antibacterial activity, wound healing, and other biomedical uses [28]. The swelling ratio of a porous deproteinized natural rubber film loaded with silver nanoparticles is shown in Figure 2a. A porous deproteinized natural rubber film demonstrated a low swelling ratio, according to earlier research [29]. The swelling ratio of the deproteinized natural rubber film was not significantly affected by the loading of silver nanoparticles in porous deproteinized natural rubber film. The swelling ratio of the deproteinized natural rubber film demonstrated that the polymer matrix may absorb water during the duration of the investigation. The produced film’s poor absorptive capacity is attributable to the fact that deproteinized natural rubber film is a complex substance, resulting in a decrease in water absorption due to the presence of molecules that may produce crosslinking between polymer chains, reducing water ingress into the structure [30].

The deproteinized natural rubber film showed no erosion; however, the porous deproteinized natural rubber film loaded with silver nanoparticles showed a significant tendency of increasing the film’s erosion as the amount of silver nanoparticles in the film increased (Figure 2b). This occurred because the silver nanoparticles were removed from the film after the film had fully swollen. However, when PN04 and PN05 were compared, the erosion of the film was not significantly different, illustrating that the amount of silver nanoparticles in the film was not significant. The benefits of increased erosion have been described for rhodamine B-loaded transdermal patches by adding poly(vinyl alcohol) to the deproteinized natural rubber latex for a significant increase in drug release [15]. 

### 3.5. Moisture Vapor Transmission Rate

**Figure 3:**
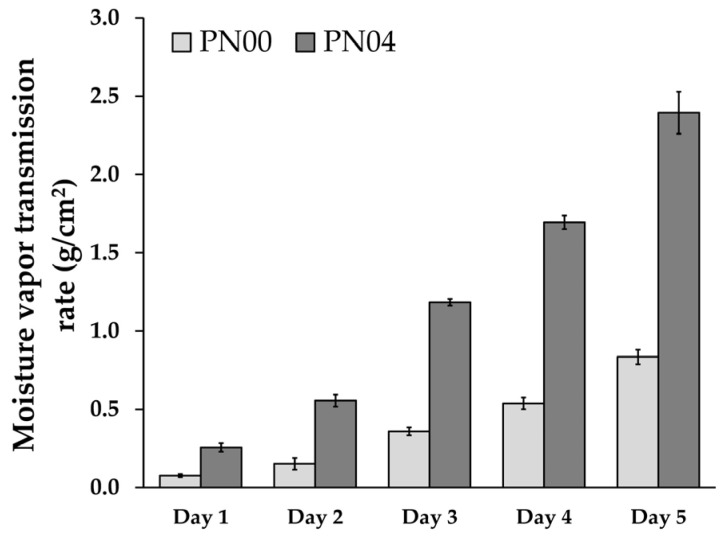
Moisture vapor transmission rate of porous deproteinized natural rubber film loaded with silver nanoparticles.

The PN04 was the appropriate and best preparation for porous deproteinized natural rubber film loaded with silver nanoparticles. The moisture vapor transmission rate of the porous deproteinized natural rubber film loaded with silver nanoparticles (PN04) is shown in Figure 3. The water vapor transmission of the porous deproteinized natural rubber film loaded with silver nanoparticles was greater than that of pure porous deproteinized natural rubber film. The film’s distinct characteristics were identified after fabrication and confirmed by mechanical evaluation, swelling ratio, and erosion. The film has a significantly high permeability to water vapor because the passage of the vapor is facilitated at the interface between the polymers due to low polymeric adhesion [31]. Similarly, the moisture vapor transmission rate increased significantly with increasing assessment days, indicating significant permeability.

The moisture vapor transmission rate of the porous deproteinized natural rubber film loaded with silver nanoparticles demonstrated an even more adherent polymer interface, correlating with the results of mechanical evaluation, swelling ratio, and erosion. The film was found to be attractive for application because of its homogeneity and more potent mechanical properties than the other preparations, which were useful for topical delivery systems.

### 3.6. Scanning Electron Microscopy/Energy Dispersive X-ray Analysis

**Figure 4:**
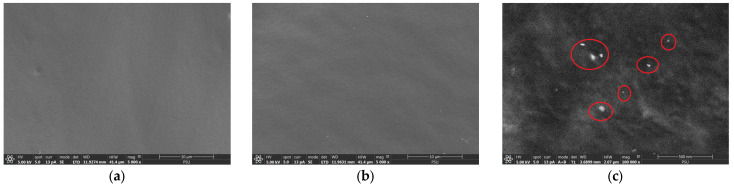
Cross-sectional morphologies of (**a**) porous deproteinized natural rubber film and (**b**) porous deproteinized natural rubber film loaded with silver nanoparticles (PN04) at 5000×, and (**c**) cross-sectional morphology of porous deproteinized natural rubber film loaded with silver nanoparticles (PN04) at 100,000×.

The SEM/EDX of the porous deproteinized natural rubber film loaded with silver nanoparticles (PN04) is shown in Figure 4. The cross-sectional morphologies of the blank porous deproteinized natural rubber film (Figure 4a) and that loaded with silver nanoparticles (Figure 4b) were both tremendously smooth. This was caused by drying during the preparation procedure to eliminate excess solvent. Therefore, it was feasible that no pores would be found in the film. However, when the film was immersed in silver nanoparticle solution, it swelled, allowing silver nanoparticles to penetrate. Due to the very low amount of silver nanoparticles present in the porous deproteinized natural rubber film (PN04 formula in Table 1), which was below the lowest level at which the technique can detect them, the EDX method was unable to determine the distribution of these silver nanoparticles. However, when the cross-sectional morphology of the porous deproteinized natural rubber film loaded with silver nanoparticles was magnified at 100,000×, some small silver nanoparticles were found in its layers (Figure 4c).

### 3.7. X-ray Diffraction

**Figure 5:**
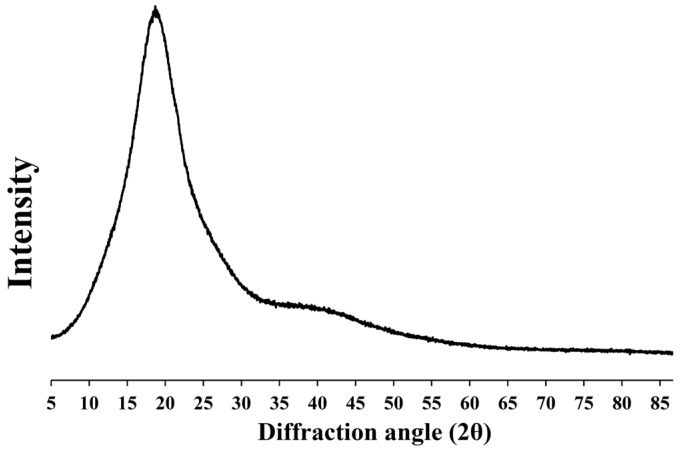
XRD pattern of porous deproteinized natural rubber film loaded with silver nanoparticles.

The crystal structure of silver nanoparticles is shown in our earlier publication [7] at 38.13, 44.11, 64.43, and 77.01°. According to the previous paper [18], we highlight the preparation of deproteinized natural rubber film and its characterization. It was discovered that the pure deproteinized natural rubber film exhibited just the broad halo spectrum, indicating a broader domain of the amorphous phase. The XRD of the porous deproteinized natural rubber film loaded with silver nanoparticles (PN04) is shown in Figure 5. These silver nanoparticle peaks were not identified in the investigated PN04 film, which had an amorphous phase similar to the pattern of deproteinized natural rubber film. This revealed that the silver nanoparticles were miscible in the porous deproteinized natural rubber film. Perhaps the silver nanoparticle content was so low that its character could not be determined using the XRD method. Furthermore, another technique such as drug content and SEM/EDX could potentially be employed to confirm the presence of silver nanoparticles in the film.

### 3.8. In Vitro Release of Silver Nanoparticles from Porous Deproteinized Natural Rubber Film

**Figure 6:**
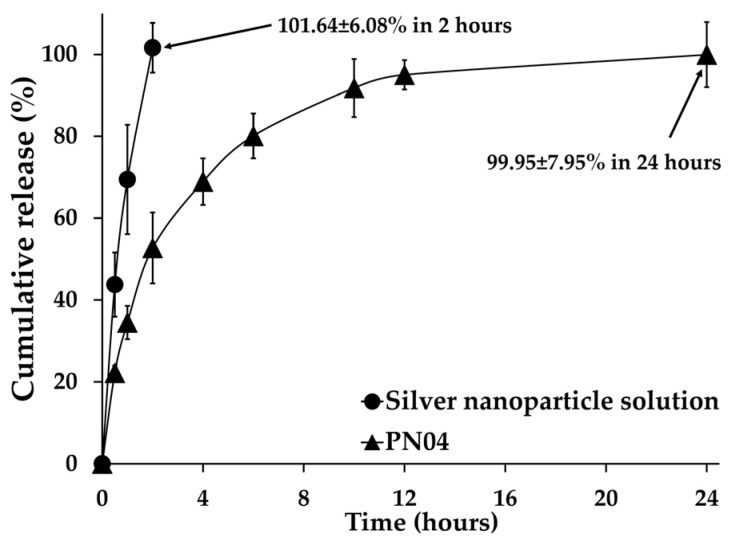
In vitro release of silver nanoparticles from porous deproteinized natural rubber film.

Figure 6 represents the in vitro release behaviors of silver nanoparticles from solution and film. The silver nanoparticles in the solution diffused and were released into the receptor media within 2 h at 101.64 ± 6.08%. This happened because the silver nanoparticles were evenly distributed in the water and transferred fast across the cellulose dialysis membrane. Similarly, the silver nanoparticles were easily released from the porous deproteinized natural rubber film. When the receptor media penetrated the matrix of the porous deproteinized natural rubber film, the matrix of the film could have subsequently swollen or eroded, causing the silver nanoparticles to diffuse or dissolve. Additionally, the porous deproteinized natural rubber film could control the release behavior of the silver nanoparticles over 24 h at 99.95 ± 7.95%. This demonstrated that the film layer had a significant effect on the release of the silver nanoparticles.

A comparison of the R^2^ values of the zero order, first order, and Higuchi models (as shown in Table 2) demonstrated that the R^2^ value of the Higuchi model was higher for the release of silver nanoparticles from the solution, but the release of silver nanoparticles from porous deproteinized natural rubber film was a first-order model. The release of the silver nanoparticles from the solution was controlled by both diffusion and dissolution processes, whereas the release of the silver nanoparticles from the porous deproteinized natural rubber film depended on the remaining silver nanoparticle content in the film.

According to the Korsmeyer–Peppas model, the diffusion exponent n-values for the release of the silver nanoparticles from the film was 0.315. Fickian diffusion was used to explain how the silver nanoparticles were released from the porous deproteinized natural rubber film because the diffusion exponent n-values were less than 0.5. Fickian diffusion occurs when the polymer relaxation time is much longer than the typical solvent diffusion period [19,32,33]. Finally, in the present investigation, the polymer matrix demonstrated the release behavior of silver nanoparticles as a diffusion-controlled process, in addition to the amount of silver nanoparticles remaining in the porous deproteinized natural rubber film.

### 3.9. In Vitro Skin Permeation of Silver Nanoparticles from Porous Deproteinized Natural Rubber Film

**Figure 7:**
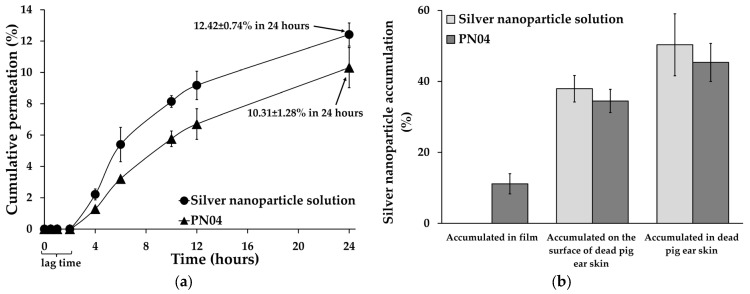
(**a**) In vitro skin permeation of silver nanoparticles from porous deproteinized natural rubber film and (**b**) silver nanoparticle accumulation in different layers.

The potential of silver nanoparticles to penetrate the dermis and epidermis of dead pig-ear skin was investigated. In vitro procedures using animal skin are frequently used to test the penetration of potentially topical drugs. The permeability parameters of the stratum corneum, which is regarded to be the rate-limiting step for skin absorption, remain unchanged after removal from the body, allowing for direct comparison [34]. The lag times for in vitro permeation of silver nanoparticles from solution and porous deproteinized natural rubber film were 2.61 and 2.66 h, respectively (Table 3), indicating the time between applying the silver nanoparticles to the skin surface (the start of the exposure) and detecting the material on the opposite side of the skin. Because the lag times for both of them were not significant, the porous deproteinized natural rubber film did not influence silver nanoparticle permeation into the skin, whereas the skin barrier alone affected silver nanoparticle permeation into the skin. After 24 h of testing, 12.42 ± 0.74% of the silver nanoparticles could pass through the dead pig-ear skin into the receptor medium for the silver nanoparticle solution, while 10.31 ± 1.28% of the silver nanoparticles were detected in the receptor medium for PN04 (Figure 7a), indicating a low level of silver nanoparticle permeation. This was because the stratum corneum acted as a barrier to silver nanoparticle permeability through the dead pig-ear skin, in contrast to the in vitro release studies [19,35]. These findings correspond with previous research, which indicated that when skin is investigated with intact tissue, fewer silver nanoparticles are identified in the receptor compartment [6,34,36]. The permeation rate (J_ss_, µg/cm^2^/h) was defined as the slope of the linear portion plot between the steady-state flux of the cumulative amount of silver nanoparticle penetration (µg/cm^2^) and time (h). The steady-state fluxes of the cumulative amount of silver nanoparticle penetration determined between 4 and 24 h for in vitro silver nanoparticle permeation from solution and porous deproteinized natural rubber film were 0.092 ± 0.010 and 0.089 ± 0.012 µg/cm^2^/h, respectively. Then, the permeability coefficient was deduced by dividing the flux by the initial silver nanoparticle loading. The permeability coefficient values of the cumulative amount of silver nanoparticles penetrating from the solution and porous deproteinized natural rubber film were 4.597 ± 0.525 × 10^−3^ and 4.267 ± 0.575 × 10^−3^ cm/h, respectively (Table 3).

Figure 7b represents the silver nanoparticle accumulation after complete in vitro skin permeation. After a 24 h in vitro permeation investigation, the amount of silver nanoparticles (from solution) that accumulated in the dead pig-ear skin was 50.32 ± 8.73%, whereas the amount of silver nanoparticles (from film) that accumulated was 45.34 ± 5.36%. The amount of silver nanoparticles (from solution) that accumulated on the surface of the dead pig-ear skin were 37.94 ± 3.74%, whereas the amount of silver nanoparticles (from film) that accumulated was 34.48 ± 3.27%. Finally, 11.15 ± 2.85% of the silver nanoparticles were detected in the porous deproteinized natural rubber film. Thus, the diffusion study revealed that for the small-sized silver nanoparticles, more nanoparticles remained localized in the skin and fewer penetrated the receptor compartment. Small nanoparticles may penetrate the skin’s stratum corneum and hair follicle orifices and reach the stratum corneum’s lowest levels while staying outside the skin’s surface, according to research on silver [34] and metallic [37] nanoparticles.

### 3.10. Antimicrobial Activity Evaluation

**Figure 8:**
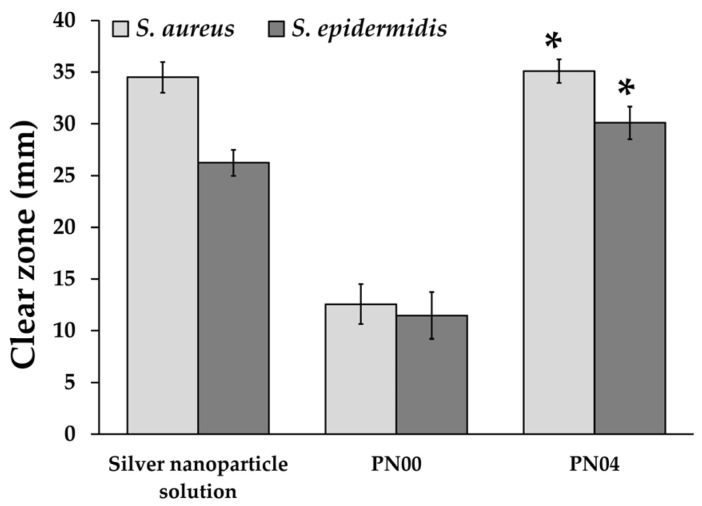
Antimicrobial activity of porous deproteinized natural rubber film loaded with silver nanoparticles (n = 3). The asterisks (*) represent significant differences (* *p* < 0.05).

Because of their greater medicinal importance, antimicrobial films continue to be of significant interest. Many synthetic polymers have been used as medical and wound dressings; however, they frequently cause skin irritations because of toxic compounds that cause negative reactions in people. Consequently, producing antimicrobial films from environmentally friendly natural sources such as biopolymers would be a superior alternative [38,39,40]. This suggests that porous deproteinized natural rubber as a natural polymer should be the first of interest in our study. Therefore, we investigated our unique film’s usefulness as an antibacterial material in this paper. The antimicrobial activities of the porous deproteinized natural rubber film loaded with silver nanoparticles are shown in Figure 8. Sterilized paper discs with and without silver nanoparticles were used as positive and negative controls, respectively. The antibacterial activity was highest in the positive control group and slowest in the negative control group. The antibacterial activity of the investigated porous deproteinized natural rubber film loaded with silver nanoparticles followed the same pattern for both microorganisms: *S. aureus* and *S. epidermidis*. The porous deproteinized natural rubber film loaded with silver nanoparticles showed high activity related to drug release. Additionally, the inhibition zones of the porous deproteinized natural rubber film loaded with silver nanoparticles (PN04) and silver nanoparticle solution were not statistically different in both *S. aureus* and *S. epidermidis* production but were significantly different from PN00 (*p* < 0.05). The smaller size of silver nanoparticles, which were more efficient for antibacterial application, might explain this finding. Additionally, the porous deproteinized natural rubber film produced a high release of silver nanoparticles (Figure 6), which had a stronger antibacterial effect. The antibacterial properties of the prepared porous deproteinized natural rubber film loaded with silver nanoparticles were interesting since doctors have expressed interest in employing topical medical products to treat wound infections. The first antibiotic therapy for infected chronic wounds is based on an empirical method. Superficial acute wounds from dermatologic treatments were historically treated with prophylactic topical antibiotics, which reduced infection rates and enhanced healing [41]. Knowing that the porous deproteinized natural rubber film loaded with silver nanoparticles demonstrated both antibacterial activities, this system might be utilized as a wound dressing for a topical film that protects the location from infection while additionally encouraging wound healing.

## 4. Conclusions

The study proves the potential for the utilization of porous deproteinized natural rubber film for topical drug delivery. The objective was to successfully achieve a porous natural rubber film loaded with silver nanoparticles by immersing the film in a solution containing silver nanoparticles. The swollen film was then dried overnight in a hot air oven set to 50 °C. Silver nanoparticles were reported to be able to get inside the film, with concentrations ranging from 7.25 to 21.03 g/cm^2^. The surface of the film had a pH of 7.00. The mechanical properties of the film with silver nanoparticle loading varied from those of the film without the loading. The film eroded more easily with the addition of silver nanoparticles; however, the swelling ratio did not change significantly. The moisture vapor transmission rate of the film was affected by increased time penetration. The silver nanoparticles were readily released from the film, although there was less permeability. The dead pig-ear skin contained a significant concentration of silver nanoparticle accumulation. The porous deproteinized natural rubber film incorporating silver nanoparticles demonstrated potent antibacterial action. Thus, the silver nanoparticle-loaded porous deproteinized natural rubber film has the potential to be utilized as a wound dressing as a topical film that induces wound healing while simultaneously protecting the region from infection.

## Figures and Tables

**Table 1:** Content of silver nanoparticles and surface pH of prepared porous deproteinized natural rubber film.

Code	Concentration of Silver Nanoparticle Solution (ppm)	Length of Immersion (Hours)	Content of Silver Nanoparticles in Porous Deproteinized Natural Rubber Film (µg/cm^2^)	Surface pH
PN00	-	-	-	7.22 ± 0.06
PN01	1000	24	7.25 ± 0.88 ^†^	7.04 ± 0.06
PN02	2000	24	12.03 ± 0.86 ^†^	7.02 ± 0.04
PN03	3000	24	16.97 ± 0.65 ^†^	7.00 ± 0.09
PN04	3000	48	20.81 ± 0.97 ^†^	7.02 ± 0.06
PN05	3000	72	21.03 ± 0.72 ^‡^	7.04 ± 0.13

^†^ There were significant differences in the silver nanoparticle content. ^‡^ There were no significant differences in the silver nanoparticle content compared to PN04.

**Table 2:** Kinetic models of in vitro release of the silver nanoparticles.

	R^2^			n	Release Rate
	Zero Order	First Order	Higuchi	Korsmeyer–Peppas	
Silver nanoparticle solution	0.9616	0.9844	0.9888	-	69.752 ± 6.083 *
Porous deproteinized natural rubber film loaded with silver nanoparticles (PN04)	0.7920	0.9902	0.9342	0.315 ± 0.044	0.334 ± 0.063 **

* Calculated from the Higuchi model. ** Calculated from the first order.

**Table 3:** Permeation parameters of silver nanoparticles across the skin from porous deproteinized natural rubber film.

	Lag Time (h)	J_ss_ (µg/cm^2^/h)	K_p_ × 10^−3^ (cm/h)
Silver nanoparticle solution	2.61	0.092 ± 0.010	4.597 ± 0.525
Porous deproteinized natural rubber film loaded with silver nanoparticles (PN04)	2.66	0.089 ± 0.012	4.267 ± 0.575

## Data Availability

Not applicable.
